# Supplementary material for: Cardiovascular deconditioning during long-term spaceflight through multiscale modeling
Source: NPJ Microgravity. 2020 Oct 1;6:27. doi: 10.1038/s41526-020-00117-5 (PMC7529778; doi:10.1038/s41526-020-00117-5)
Supplement: Supplementary file 1 — Supplementary Information [file 41526_2020_117_MOESM1_ESM.pdf]

Supplementary Information
Cardiovascular deconditioning during long-term spaceflight through multiscale modeling

C. Gallo, L. Ridolfi, S. Scarsoglio

Multiscale model

A comprehensive description of the multiscale model is presented, with Supplementary Table 1 displaying the nomenclature adopted. The equations representing each cardiovascular sub-model are summarized in the following subsections, together with the parameter values in 1G supine and 0G configurations. The multiscale model is physical-based and parameters are measure-based, so there cannot be any statistical inference or overfitting between parameters and obtained results. Parameters were given in terms of geometrical, mechanical and structural properties of the cardiovascular system (such as vessel diameter and length, resistances, compliances, inertances, etc), while raw results were expressed in terms of fluid dynamics variables, such as blood flow rates, volumes, pressures, vessel cross-sectional areas, and valve opening angles.

Arterial tree

The arterial tree consists of 48 large-to-medium sized arteries, neglecting the right leg because of the symmetry in the arterial networks of lower extremities. Large-to-medium sized arteries were solved through the 1D model proposed by Guala and co-authors [1, 2]. Mass and momentum balance equations were integrated over the arterial section, leading to the following 1D system

$$\begin{cases} \dfrac{\partial A}{\partial t} + \dfrac{\partial Q}{\partial x} = 0, \\ \dfrac{\partial Q}{\partial t} + \dfrac{\partial}{\partial x}\left(\beta \dfrac{Q^2}{A}\right) = -\dfrac{A}{\rho}\dfrac{\partial P}{\partial x} + N_4 \dfrac{Q}{A}, \end{cases} \tag{1}$$

with x the axial coordinate, t time, A the vascular section, Q the flow rate, P the pressure, β the Coriolis coefficient, ρ the blood density, and N_4 the viscous term. Since A and Q are dependent variables, a constitutive equation for pressure is introduced

$$P = B_1 + B_2 A + B_3 A^2 + B_4 A^3 - B_5 \frac{1}{\sqrt{A}} \frac{\partial Q}{\partial x}, \tag{2}$$

where the coefficients B_i, for $i = 1 : 5$, contain all the information about the regional mechanical properties of the arterial walls.

A more detailed presentation of the 1D model was provided in previous studies [1–3], while geometrical data of the arterial tree and characteristic impedances of terminal arteries are reported in Supplementary Table 2.

Peripheral, venous and pulmonary circulation

Arterioles, capillaries, venules, veins, pulmonary arteries and veins were all reproduced through RLC electric circuits [4–6]. The RLC circuit for the generic j-th compartment was resolved as follows

$$\begin{cases} \dfrac{\mathrm{d}P_j}{\mathrm{d}t} = \dfrac{Q_{j-1} - Q_j}{C_j}, \\ \dfrac{\mathrm{d}Q_j}{\mathrm{d}t} = \dfrac{P_j - R_j Q_j - P_{j+1}}{L_j}, \\ V_j = V_{0,j} + C_j P_j. \end{cases} \tag{3}$$

In this latter, V_j and $V_{0,j}$ are the total and unstressed volumes associated to the compartment, Q_{j-1} and Q_j are the flow rates entering and exiting the compartment, and P_j and P_{j+1} are the pressures at the inlet and outlet of the compartment. Resistances, R_j, inertances, L_j, and compliances, C_j, are proper of each compartment and chosen according to [5, 6] with adjustments as indicated in Supplementary Table 3. Unstressed volumes of all compartments, provided as well in Supplementary Table 3, were chosen in order to make a total blood volume of 5.5 l and respect the physiological distribution of blood within both vascular and body regions [7, 8] for a healthy subject in supine condition.

Cardiac chambers

Similarly to what proposed by other authors [4–6], the dynamics of the cardiac chambers were mimicked through a time-varying elastance model as

$$P_{ch} = E_{ch}(V_{ch} - V_{0,ch}), \tag{4}$$

This relation links the chamber pressure, P_{ch}, to the stressed chamber volume, $(V_{ch} - V_{0,ch})$, through the elastance function, $E_{ch} = E_{A,ch} e_{ch} + E_{B,ch}$. $E_{B,ch}$ and $E_{A,ch}$ are the minimal and amplitude values of the elastance, respectively, e_{ch} is a normalized time-varying function of the elastance having a different expression for atria and ventricles. Atrial, e_a, and ventricular, e_v, functions were determined as indicated by [4–6], namely

$$e_a = \begin{cases} \dfrac{1}{2}\left[1 + \cos\left(\pi \dfrac{(t + RR - t_{ar})}{T_{ar}}\right)\right] & 0 \le t \le t_{ar} + T_{ar} - RR, \\ 0 & t_{ar} + T_{ar} - RR < t \le t_{ac}, \\ \dfrac{1}{2}\left[1 - \cos\left(\pi \dfrac{(t - t_{ac})}{T_{ac}}\right)\right] & t_{ac} < t \le t_{ac} + T_{ac}, \\ \dfrac{1}{2}\left[1 + \cos\left(\pi \dfrac{(t - t_{ar})}{T_{ar}}\right)\right] & t_{ac} + T_{ac} < t \le RR, \end{cases} \tag{5}$$

and

$$e_v = \begin{cases} \frac{1}{2}\left[1 - \cos\left(\pi \frac{t}{T_{vc}}\right)\right] & 0 \le t \le T_{vc}, \\ \frac{1}{2}\left[1 + \cos\left(\pi \frac{(t - T_{vc})}{T_{vr}}\right)\right] & T_{vc} < t \le T_{vc} + T_{vr}, \\ 0 & T_{vc} + T_{vr} < t \le T, \end{cases} \tag{6}$$

where T_{ac}/T_{ar} and T_{vc}/T_{vr} are the periods of contraction/relaxation for atria and ventricles, t_{ac} and t_{ar} are the times when atria start contracting and relaxing, respectively. Characteristic times of the e_a and e_v functions, $E_{A,ch}$, $E_{B,ch}$, and $V_{0,ch}$ values were taken as suggested in [4–7] with adjustments and are listed in Supplementary Table 4.

Cardiac valves

The motion of each cardiac valve was adequately modeled through the pressure-flow relation presented by Blanco et al. [4, 9], namely

$$\begin{cases} L_v \frac{dQ_v}{dt} + R_v Q_v + B_v |Q_v| Q_v = \frac{(1 - \cos(\theta_v))^4}{(1 - \cos(\theta_{max}))^4} (P_1 - P_2), \\ I_{ao} \frac{d^2\theta_v}{dt^2} = F_{pr} + F_{bm} - F_{fr} - F_{vo}. \end{cases} \tag{7}$$

This system leads to the approximation of the valvular flow rate based on the fluid inertance, L_v, the viscous resistance, R_v, the turbulent flow separation coefficient, B_v, the pressure difference across the valve $P_1 - P_2$, and the degree of the valve opening angle, θ_v, that is, between 0 and θ_{max}=75°. θ_v was calculated based on the rotational inertia (I_{ao}) and the forces applied on the valve leaflets, that is, the pressure gradient, F_{pr}, the dynamic action of the blood pushing on the blood leaflets, F_{bm}, the frictional effects caused by the tissue resistance, F_{fr}, and the role of the vortexes downstream of the valve, F_{vo}. Forces applied on the valve leaflets and related parameters were set as in [4].

Baroreflex model

A short-term regulation of the arterial pressure was guaranteed by the baroreflex model presented by Ottesen et al. [10]. At the end of each heartbeat, the mean aortic-carotid sinus pressure, p_{acs}, was evaluated as the arithmetic average of the aortic, right- and left-carotid sinus pressures, each averaged over the previous heartbeat duration, as suggested by [11]. Sympathetic (n_s) and parasympathetic (n_p) responses were evaluated as

$$n_s(p_{acs}) = \frac{1}{1 + \left(\frac{p_{acs}}{p_{acs,ref}}\right)^{\nu}}, \qquad n_p(p_{acs}) = \frac{1}{1 + \left(\frac{p_{acs}}{p_{acs,ref}}\right)^{-\nu}}, \tag{8}$$

with ν determining the steepness of the sympathetic and parasympathetic activities, and $p_{acs,ref}$ equal to the reference aortic-carotid sinus pressure. In 1G supine condition ν=7 and the baseline steady-state values are $p_{acs,ref}$=92.61 mmHg and HR=75 bpm,

as set in [10]. Reactions of efferent organs were advanced in time through the ordinary differential equation

$$\frac{dx_k}{dt} = \frac{1}{\tau_k}\left(-x_k + \alpha_k n_s(p_{acs}) - \beta_k n_p(p_{acs}) + \gamma_k\right), \tag{9}$$

with x_k the k-th efferent organ, α_k and β_k the weights of the sympathetic and parasympathetic activities, and γ_k equal to x_k when n_s=n_p. Efferent organs are the heart rate (HR), the amplitude values of left- and right-ventricular elastance ($E_{A,lv-rv}$), arteriolar and capillary resistances (R_{art-c}), compliances and unstressed volumes of venules and veins (C_{ven-v} and $V_{0,ven-v}$). Parameters of the baroreflex model reported in Supplementary Table 5 were set as in [10], with adjustments suggested as in [11, 12]. Baseline steady-state values for $E_{A,lv-rv}$, R_{art-c}, C_{ven-v}, and $V_{0,ven-v}$ in 1G and 0G conditions are provided in Supplementary Tables 3 and 4. Values at 1G are set in Supplementary Table 3 following [5, 6] with adjustments, and in Supplementary Table 4 following [4–7]. Values at 0G are explained in the "Spaceflight setting: cardiovascular mechanisms and adopted changes" Section.

Numerical method

The mathematical model was solved through a two-steps Runge-Kutta Discontinuous-Galerkin approach. In particular, space was discretized by a Discontinuous-Galerkin scheme, while temporal advancement was managed through a two-steps Runge-Kutta method. Boundaries between 1D elements (arterial bifurcations) or 1D and 0D models (aortic valve and arterial characteristic impedances) were solved by combining physical and compatibility conditions. Physical conditions were represented by the conservation of mass and total pressure at arterial junctions, system (4) for aortic valve, and the constitutive equation of the characteristic impedance at the interface between arteries and arterioles. Compatibility conditions derived from the quasi-linear form of the system solving 1D arteries (1,2) and allowed us to link A and Q at terminal arteries.

Definition of cardiac parameters

End-systolic left ventricular volume, V_{lves} [ml], is the left ventricle volume at the closure of the aortic valve, end-diastolic left ventricular volume, V_{lved} [ml], corresponds to the closure of the mitral valve. Stroke volume is defined as $SV = V_{lved} - V_{lves}$ [ml], ejection fraction is $EF = SV/V_{lved} \cdot 100\%$ [%]. Cardiac output is $CO = SV \cdot HR$ [l/min], stroke work, SW/min [J/min], is measured as the area within the left ventricle pressure-volume loop per beat. $P_{ca,syst}$ [mmHg] and $P_{ca,dias}$ [mmHg] are the systolic and diastolic central aortic pressures, respectively. The mean arterial pressure is estimated as $MAP = \frac{2}{3}P_{ca,dias} + \frac{1}{3}P_{ca,syst}$. Oxygen consumption is estimated through: (i) tension time index per minute, $TTI/min = \overline{P}_{lv} \cdot RR \cdot HR$ [mmHg s/min], where $\overline{P}_{lv}$ is the mean value of the left ventricular pressure, P_{lv}, over a cardiac beat; (ii) and the rate pressure product, $RPP = P_{ca,syst} \cdot HR$ [mmHg/min]. The augmentation index (AI) is the ratio between the augmented pressure (AP) - which is the difference between systolic pressure and the first systolic shoulder caused by wave reflection - and the pulse pressure (PP), being this latter the difference between systolic and diastolic pressures [13]. Both AI and PP are evaluated for the ascending aortic pressure, namely AI_{AA} and PP_{AA}.

Spaceflight setting: cardiovascular mechanisms and adopted changes

The cardiovascular changes due to weightlessness included into the model are fully described. The 0G setting was based on a comprehensive bibliographic investigation, where more than fifty studies have been considered. As mentioned in the Main Text, more recent studies of long-term spaceflights with customary countermeasures have been preferred, by paying less attention to the others, such as ground-based and simulated microgravity experiments [14–22], short-term spaceflights [23–30], older studies [14, 15, 31–35], spaceflights with declared regular countermeasures [36–39], studies with different baseline posture [26] and focused on transient non-steady state response [26–28, 40–42].

Blood shift

We focused on blood shift only, since the present model is able to actively handle variations of blood in volume and distribution. Effects of interstitial fluid shift and muscle atrophy were intrinsically taken into account in the overall setting of the long-term spaceflight configuration, in particular by modifying the total and unstressed volumes of all the cardiovascular compartments, as cleared in the next paragraphs.
To evaluate blood shift from lower to upper body we need to define: (i) the reference shift point, and (ii) the amount of blood volume.

(i) The volume indifference point (VIP), defined as the body region where blood volume does not change as posture varies, individuates the reference point for the fluid shift and determines the partition between upper and lower body [43]. Although there is an intersubject variability, VIP is usually located in the abdominal region. It was observed that each cardiovascular region experiences a blood volume variation due to posture which increases (in absolute terms) with the distance between the VIP and the center of mass of the region itself [44]. Moreover, the severity of orthostatic intolerance was related to the VIP position [43]. We here set the VIP at the abdominal level, between the epigastric and mesogastric planes, according to the most common VIP position which is typically located at about 65% of an individuals height [44]. In so doing, the lower body includes the legs and the lower abdomen (hypogastric and mesogastric planes), while the upper body consists of the upper abdomen (epigastric plane), the thoracic-cardiac region, head and arms (see Fig. 5).

(ii) To estimate the amount of blood shift, ground-based experiments (e.g., water immersion and bed rest studies) were not fully exploitable: they alter but do not eliminate the hydrostatic pressure gradient, also introducing side effects such as fluid movement from interstitial to intravascular space and abnormal mechanoreceptor stimulation due to the contact with bed [16–19, 21, 32, 45]. Measurements from space missions showed that both legs, with respect to the upright position in 1G, lose 1200-1400 ml during the first day in microgravity and up to 2 l after 4-5 days [32, 33, 35, 46]. However, these data rather than to blood shift referred to fluid shift, which is ruled by different other midterm transient mechanisms, such as interstitial fluid shift, blood volume reduction, and muscle atrophy [33, 47]. Given the substantial lacking of definitive data on blood shift, we estimated it from lower limbs data measured during parabolic flight. We reasonably assumed that the short term exposure to microgravity (20-30 [s] per session) only induces blood shift, without promoting the above midterm mechanisms acting on larger temporal scales (minutes to hours). For blood shift of other

regions, in particular lower abdomen and upper body, considerations based on the vessel distensibility and the distance from VIP were combined together and referred to the legs data.
Lower body
From parabolic flight we managed to infer that during microgravity both legs lose about 330 ml of blood with respect to the upright position in 1G [47, 48]. Considering that in 1G blood volume of both legs is about 900 ml in supine position [8] and 1265 ml in upright position [49, 50], we plausibly supposed that blood shift is directly proportional to the reference 1G condition (either upright or supine). We obtained a blood shift of 235 ml for both legs with respect to the 1G supine condition.
To estimate lower abdomen blood shift, we accounted for both compliance and distance from VIP values, taking legs data as reference. Lower abdomen is a bit more compliant than lower limbs (17.28 vs 10.42 ml/mmHg, estimated by [6]), but closer to VIP than legs (14.70 vs 44.10 cm, center of mass distances, [51]). By equally weighting the two contributes and assuming a direct proportionality with respect to the blood shift from the legs, we achieved a blood shift from lower abdomen of about 234 ml.

The overall computed blood shift from lower body was about 469 ml with respect to the 1G supine condition. If referred to the 1G upright condition - considering that blood shift from legs is 330 ml [47, 48] and from lower abdomen with the above adopted criteria is 329 ml - we would have attained an overall lower body blood shift of 659 ml. This value is close to the 700 ml found as the most reliable estimate of lower body blood shift in microgravity with respect to the 1G upright condition [32, 33]. The good agreement is an *a posteriori* validation of the realistic blood shift here proposed.
Upper body
We supposed that the 469 ml of blood leaving the lower body are distributed in the upper body with the same above criterion, which equally combines vessel compliance and distance from VIP: regions more compliant and further from VIP proportionally receive more blood volume. By dividing the upper body into three main regions (head-arms: C=15.63 ml/mmHg, d=24.20 cm; cardiac-thoracic: C=50.12 ml/mmHg, d=24.20 cm; upper abdomen: C=50 ml/mmHg, d=7.85 cm, [6, 51]), the head-arms, cardiac-thoracic, and upper abdomen regions received 133, 202, and 134 ml of blood, respectively.

Blood volume reduction

Blood volume reduction occurs during sustained presence in microgravity [52–54], starting from the very beginning (17% plasma reduction was observed on the first day) and completing within 6 weeks [45, 55]. Different literature data indicated the decrease of total blood volume in the range between 9% and 15% [55–59]. Accordingly, we set the blood volume percentage variation around the average value of the above interval, namely -11.5%. Within each region, total volumes, V, were reduced proportionally to the configuration after blood shift. Unstressed volumes V_0 were set to preserve V_0/V as in the configuration after blood shift only.

Cardiac function

Long-term data regarding cardiac function are conflicting mainly due to the presence of a strict countermeasure protocol. If countermeasures were substantially absent or not regularly followed, cardiac dysfunction and atrophy started to emerge [56, 57, 60]. The adoption of an intense exercise program was able to most contrast cardiac atrophy [61, 62] and limit cardiac dysfunction [39]. In any case, the reduction of contractility

and cardiac function was quantified by the decrease of V_{lved} between 5% and 13% [39, 56, 57, 63, 64], SV between 14% and 23% [39, 56, 57, 63], and EF between 5% and 11% [39, 56, 57, 63, 64]. Given the rapid postflight cardiac recovery, these variations seemed not to be related to structural cardiac changes, but plausibly related to a cardiac remodeling induced by an intrathoracic overpressure, which in turn leads to cardiac volume and compliance variations [56].

In absence of precise indications in literature, we based elastance variations in agreement with the observed reduction of the contractile indexes and plausibly assumed a percentage variation similar to the one observed for the leg venous compliance (see next Section). Amplitudes of left ventricular ($E_{A,lv}$) and right ventricular ($E_{A,rv}$) elastances were therefore decreased by 27%. Minimum left and right ventricular elastance values ($E_{B,lv}$ and $E_{B,rv}$, respectively) were increased by 3% with respect to the 1G supine condition, to accommodate contractile reduction and considering that minimum values are related to the diastolic phase, which is mildly involved in defining the contractile capability (i.e., percentage variation about ten-fold lower). Given the lack of definitive data on the right heart, we assumed for the right ventricle the same percentage variations adopted for the left ventricle. To account for the increased intrathoracic pressure without modifying VIP position, pulmonary arteries and veins compliances were increased by 4% and 5%, respectively. The good validation results obtained for contractile indexes reported in the Main Text *a posteriori* support the adopted parameter choice.

In the long-term cardiac volume reduction was found beetween -8% and -14% with respect to preflight [39, 54]. We took a 10% cardiac volume reduction, which is an intermediate value within the indicated range, with respect to 1G supine condition. This variation was obtained by decreasing the unstressed volume of all cardiac chambers by 90% with respect to 1G supine condition.

Legs venous compliance

Although few contrasting results did not highlight any variation [21, 30], different studies showed an increase of the legs venous compliance after long-term exposure to simulated or real microgravity [14, 15, 22, 64–67]. Recent outcomes of long-term spaceflights were the most reliable ones and attested this change between +25% and +33.33% [64–66]. We considered an increase of the legs venous compliance by 27% with respect to 1G supine condition.

Arterial resistances

Long-term exposure to microgravity leads to a reduction of the lower limbs resistances and an increase of the cerebral resistances, with respect to the preflight condition. In particular, it has been observed a decrease of the renal resistance by 15% [63, 68, 69] and of the femoral resistance between 5% and 12% [39, 63, 64], after 4-6 months of spaceflight. Cerebral resistances increased between 5% and 15% [39, 63, 68, 69]. Moreover, all arterial resistances below the heart level were supposed to drop during spaceflight due to the missing gravitational stimuli [64]. According to this and without further indications on specific sites, we reduced all arterial resistances of the lower body (below VIP) by 10% with respect to 1G supine condition. On the contrary, we increased resistances of vertebral and carotid arteries by 10%, which is the average value of the observed range of variations.

Baroreflex response

Since our goal is to reproduce the long-term spaceflight configuration in steady state condition, we only considered variations related to the baseline steady-state values of baroreceptor mechanisms.

Chronotropic effects were observed in weightlessness depending on the mission length and the adoption of countermeasures. During long-term spaceflight, HR was found to remain constant or either increase. The great variability of results is mostly due to the fact that variations were often referred to either sitting/upright or supine 1G conditions (recall that in 1G upright condition HR is up to +24% than in 1G supine condition [37]). However, we focused on variations with respect to the 1G supine condition and HR increased from 5% to 15% [34, 36–39, 56, 57]. We set the HR baseline steady-state value equal to 84.75 bpm, as increased by 13% with respect to the 1G supine condition.

The mean arterial pressure, MAP, in long-term spaceflight resulted in a reduction between 2% and 10%, with respect to 1G supine condition [36–39]. The smallest reductions occurred when a specific countermeasure protocol was adopted. Since our aim is to study spaceflight variations without *ad hoc* countermeasures, we set the baseline steady-state value of the average aortic-carotid sinus pressure $p_{acs,ref}$ equal to 83.28 mmHg, as decreased by 10% with respect to the 1G supine condition.

Symbol	Description	Symbol	Description
VIP	Volume indifference point	C_v	Venous compliance
HA	Head and arms	R_v	Venous resistance
U ABD	Upper abdominal	L_v	Venous inertance
L ABD	Lower abdominal	C_{vc}	Vena cava compliance
LEGS	Legs	R_{vc}	Vena cava resistance
SVC	Superior vena cava	L_{vc}	Vena cava inertance
IVC	Inferior vena cava	E_{ra}	Right-atrial elastance
RH	Right heart	E_{rv}	Right-ventricular elastance
PC	Pulmonary circulation	E_{la}	Left-atrial elastance
LH	Left heart	E_{lv}	Left-ventricular elastance
RA	Right atrium	R_{tv}	Tricuspid valve resistance
TV	Tricuspid valve	L_{tv}	Tricuspid valve inertance
RV	Right ventricle	R_{pv}	Pulmonary valve resistance
PV	Pulmonary valve	L_{pv}	Pulmonary valve inertance
P ART	Pulmonary arteries	C_{pa}	Pulmonary arteries compliance
P VEN	Pulmonary veins	R_{pa}	Pulmonary arteries resistance
LA	Left atrium	C_{pv}	Pulmonary veins compliance
MV	Mitral valve	R_{pv}	Pulmonary veins resistance
LV	Left ventricle	R_{mv}	Mitral valve resistance
AV	Aortic valve	L_{mv}	Mitral valve inertance
Z_i	Characteristic impedance	R_{av}	Aortic valve resistance
C_{art}	Arteriolar compliance	L_{av}	Aortic valve inertance
R_{art}	Arteriolar resistance	p_{acs}	Aortic-carotid sinus pressure
L_{art}	Arteriolar inertance	HR	Heart rate
C_c	Capillary compliance	n_s	Sympathetic signal
R_c	Capillary resistance	n_p	Parasympathetic signal
L_c	Capillary inertance	$E_{A,rv}$	RV elastance amplitude
C_{ven}	Venular compliance	$E_{A,lv}$	LV elastance amplitude
R_{ven}	Venular resistance	$V_{0,ven}$	Unstressed venular volume
L_{ven}	Venular inertance	$V_{0,v}$	Unstressed venous volume

Supplementary Table 1: Legend of the multiscale model (see Fig. 5 of the Main Text).

**N** (Right/Left)	**Arterial segment**	ℓ [mm]	D_{in} [mm]	D_{out} [mm]	Z [mmHg s/ml]
1	Ascending aorta	40	29.40	28.80	
2	Aortic arch A	20	24.10	24	
3	Brachiocephalic	34	19.39	18	
4/19	Subclavian A	34	12.88/11	9/8.5	
5/15	Common carotid	94/139	15.12/12.36	7/6	
6/20	Vertebral	149/148	4.07/3.85	2.80/2.80	11.72/11.72
7/21	Subclavian B, axillary, brachial	422/422	8.91/8.42	4.70/4.70	
8/22	Radial	235/235	3.70/3.29	3.10/2.80	9.27/11.72
9/23	Ulnar A	67/67	3.70/4.04	3.40/4.04	
10/24	Interosseous	79/79	2.10/1.80	1.80/1.80	32.37/32.37
11/25	Ulnar B	171/171	3.20/4.10	2.80/3.70	11.72/6.17
12/16	Internal carotid	178/178	5.70/5.04	4.30/4.10	4.37/4.87
13/17	External carotid	41/41	5/4.47	4.50/4.10	3.93/4.87
14	Aortic arch B	39	22.04	20.80	
18	Thoracic aorta	52	20	18.90	
26	Intercostals	80	12.60	9.50	0.71
27	Thoracic aorta B	104	16.50	12.90	
28	Abdominal aorta A	53	12.20	12.20	
29	Celiac A	20	7.80	6.90	
30	Celiac B	25	5.20	4.90	
31	Hepatic	66	5.40	4.40	4.14
32	Gastric	71	3.20	3	7.09
33	Splenic	63	4.20	3.90	5.47
34	Superior mesenteric	59	7.90	7.10	1.38
35	Abdominal aorta B	20	11.50	11.30	
36/38	Renal	32/32	4.94/4.94	4.94/5.2	3.17/2.82
37	Abdominal aorta C	20	11.21	11.21	
39	Abdominal aorta D	106	11.02	11	
40	Inferior mesenteric	50	4.70	3.20	8.62
41	Abdominal aorta E	20	10.80	10.40	
42	Common iliac	59	7.90	7	
43	Inner iliac	50	4	4	5.16
44	External iliac	144	6.40	6.10	
45	Deep femoral	126	4	3.70	6.17
46	Femoral	443	5.20	3.80	
47	Anterior tibial	343	2.60	2.30	10.20
48	Posterior tibial	321	3.10	2.80	11.72

Supplementary Table 2: Geometric and material data of the arterial tree: arterial segment length, ℓ, proximal and distal lumen diameters, D_{in} and D_{out}, characteristic impedances, Z, of terminal arteries. Z is calculated as $\rho PWV/A_{out}$, where $\rho = 1050$ kg/m^3 is the blood density, PWV is the pulse wave velocity, and A_{out} is the distal area. First column (N) refers to the numbering adopted in Fig. 5 of the Main Text.

**Cardiovascular region**	**1G R, C, L, V_0**	**0G R, C, L, V_0**
	Arteries	
Pulmonary	0.08, 3.8, /, 42.80	0.08, 3.97, /, 58.24
	Arterioles	
Vertebral (6/20)	25.88, 0.013, 0.019, 4	28.40, 0.013, 0.019, 2.85
Radial (8/22)	17.03, 0.014, 0.018, 3.40	17.03, 0.014, 0.018, 2.25
Interosseous (10/24)	393.70, 0.0009, 0.07, 3.40	393.70, 0.0009, 0.07, 2.25
Ulnar B (11/25)	19.69, 0.014, 0.018, 3.40	19.69, 0.014, 0.018, 2.25
Internal carotid (12/16)	23.60, 0.015, 0.017, 4	25.90, 0.015, 0.017, 2.85
External Carotid (13/17)	21.85, 0.015, 0.017, 4	23.98, 0.015, 0.017, 2.85
Intercostals (26)	5.61, 0.054, 0.009, 6.60	5.61, 0.054, 0.009, 5.45
Hepatic (31)	16.24, 0.021, 0.015, 29.60	16.24, 0.021, 0.015, 28.45
Gastric (32)	8.91, 0.033, 0.012, 8.90	8.91, 0.033, 0.012, 7.75
Splenic U (33)	127.98, 0.014, 0.11, 11.20	127.98, 0.014, 0.11, 10.05
Splenic L (33)	25.60, 0.014, 0.022, 11.20	23.04, 0.014, 0.022, 10.05
Superior mesenteric (34)	3.85, 0.081, 0.007, 15.40	3.46, 0.081, 0.007, 14.25
Renal U (36/38)	8.62, 0.068, 0.016, 5.70	8.62, 0.068, 0.016, 4.55
Renal L (36/38)	8.62, 0.068, 0.016, 5.70	7.76, 0.068, 0.016, 4.55
Inferior mesenteric (40)	30.74, 0.011, 0.02, 11.50	27.60, 0.011, 0.02, 10.35
Inner iliac (43)	23.48, 0.014, 0.018, 3.4	21.08, 0.014, 0.018, 2.25
Deep femoral (45)	13.37, 0.023, 0.014, 13.90	12, 0.023, 0.014, 12.75
Anterior tibial (47)	5.51, 0.023, 0.014, 13.90	4.95, 0.023, 0.014, 12.75
Posterior tibial (48)	30.44, 0.010, 0.021, 13.90	27.33, 0.010, 0.021, 12.75
	Capillaries	
HA	0.81, 0.03, 0.00045, 72.20	0.81, 0.03, 0.00045, 75
U ABD	0.65, 0.031, 0.00043, 89.80	0.65, 0.031, 0.00043, 89.58
L ABD	0.68, 0.038, 0.00047, 48.10	0.68, 0.038, 0.00047, 27.33
LEGS	0.87, 0.032, 0.00034, 57.20	0.87, 0.032, 0.00034, 35.49
	Venules	
HA	0.26, 0.6, 0.0005, 278.40	0.26, 0.6, 0.0005, 289.21
U ABD	0.21, 0.61, 0.00077, 256.30	0.21, 0.61, 0.00077, 255.68
L ABD	0.22, 0.46, 0.00013, 226.60	0.22, 0.46, 0.00013, 128.77
LEGS	0.28, 0.53, 0.00061, 269.80	0.28, 0.53, 0.00061, 167.40
	Veins	
HA	0.022, 15, 0.00056, 312	0.022, 15, 0.00056, 324.11
SVC	5E-4, 5, 0.0005, 14.30	5E-4, 5, 0.0005, 14.27
IVC	5E-4, 15, 0.0005, 4.80	5E-4, 15, 0.0005, 4.99
Pulmonary	0.01, 20.5, /, 224.60	0.01, 21.47, /, 305.60
U ABD	0.035, 49.35, 0.00084, 427.60	0.035, 49.35, 0.00084, 426.57
L ABD	0.06, 16.78, 0.00094, 412	0.06, 16.78, 0.00094, 234.13
LEGS	0.07, 9.87, 0.00067, 490.60	0.07, 12.53, 0.00067, 304.39

Supplementary Table 3: Parameters of the 0D compartments: resistance R [mmHg s/ml], compliance C [ml/mmHg], inertance L [mmHg s^2/ml], and unstressed volumes V_0 [ml], in 1G supine and 0G conditions.

	**RA**	**RV**	**LA**	**LV**
T_{ac} **[s]**	0.17 RR		0.17 RR	
T_{ar} **[s]**	T_{ac}		T_{ac}	
T_{vc} **[s]**		0.3 $\sqrt{RR}$		0.3 $\sqrt{RR}$
T_{vr} **[s]**		T_{vc}/2		T_{vc}/2
t_{ac} **[s]**	0.8 RR		0.8 RR	
t_{ar} **[s]**	$t_{ac} + T_{ac}$		$t_{ac} + T_{ac}$	
E_A **[mmHg/ml]**	0.08	0.55 (1G), 0.4015 (0G)	0.07	2.75 (1G), 2.0075 (0G)
E_B **[mmHg/ml]**	0.09	0.06 (1G), 0.0616 (0G)	0.09	0.07 (1G), 0.0719 (0G)
$V_{0,ch}$ **[ml]**	6 (1G), 0.6 (0G)	12 (1G), 1.2 (0G)	6 (1G), 0.6 (0G)	7 (1G), 0.7 (0G)

Supplementary Table 4: Heart parameters: periods of contraction and relaxation for atria (T_{ac} and T_{ar}) and ventricles (T_{vc} and T_{vr}), minimum and amplitude values of elastance ($E_{B,ch}$ and $E_{A,ch}$), and unstressed volumes ($V_{0,ch}$) for each cardiac chamber.

**Efferent Organ**	τ **[s]**	α	β	γ	**min**	**max**
HR	3	0.75	0.75	1	0.25	1.75
$E_{A,lv-rv}$	3	0.40	0	0.80	0.8	1.2
R_{art-c}	15	0.80	0	0.60	0.6	1.4
C_{ven-v}	30	-0.20	0	1.10	0.9	1.1
$V_{0,ven-v}$	30	-0.42	0	1.21	0.79	1.21

Supplementary Table 5: Baroreflex parameters adopted to simulate the responses of the efferent organs, with α, β, γ, and saturation levels (max and min) are given as percentages of the baseline steady-state values.

References

[1] Guala, A., Camporeale, C., Tosello, F., Canuto, C. & Ridolfi, L., Modelling and subject-specific validation of the heart-arterial tree system, *Ann. Biomed. Eng.* **43**, 222–237 (2014).

[2] Guala, A., Camporeale, C. & Ridolfi, L., Compensatory effect between aortic stiffening and remodelling during ageing, *PLoS One* **10**, e0139211 (2015).

[3] Scarsoglio, S., Gallo, C. & Ridolfi, L., Effects of atrial fibrillation on the arterial fluid dynamics: a modelling perspective, *Meccanica* **53**, 3251-3267 (2018).

[4] Blanco, P.J. & Feijóo RA: A dimensionally-heterogeneous closed-loop model for the cardiovascular system and its applications, *Med. Eng. Phys.* 35, 652–667 (2013).

[5] Liang, F., Takagi, S., Himeno, R. & Liu, H.: Multi-scale modeling of the human cardiovascular system with applications to aortic valvular and arterial stenoses, *Med. Biol. Eng. Comp.* **47**, 743–755 (2009).

[6] Liang, F.Y., Takagi, S., Himeno, R. & Liu, H., Biomechanical characterization of ventricular-arterial coupling during aging: a multi-scale model study, *J. Biomech.* **42**, 692704 (2009).

[7] Heldt, T., Shim, E.B., Kamm, R.D. & Mark, R.G., Computational modeling of cardiovascular response to orthostatic stress, *J. Appl. Physiol.* **92**, 1239–1254 (2002).

[8] Leggett, R.W. & Williams, L.R., Suggested reference values for regional blood volumes in humans, *Health Phys.* **60**, 139–154 (1991).

[9] Korakianitis, T. & Shi, Y., Numerical simulation of cardiovascular dynamics with healthy and diseased heart valves, *J. Biomech.* **39**, 1964–1982 (2006).

[10] Ottesen, J., Olufsen, M. & Larsen, J., *Applied mathematical models in human physiology*, Society for industrial and Applied Mathematics, Philadelphia (2004).

[11] Blanco, P.J., Trenhago, P.R., Fernandes, L.G. & Feijóo, R.A., On the integration of the baroreflex control mechanism in a heterogeneous model of the cardiovascular system, *Int. J. Numer. Method Biomed. Eng.* **28**, 412–433 (2012).

[12] Gallo, C., Ridolfi, L. & Scarsoglio, S., A closed-loop multiscale model of the cardiovascular system: application to heart pacing and open-loop response, *IFMBE Proceedings* **76**, 577–585 (2020).

[13] Westerhof, N., Stergiopulos, N., Noble, M.I.M., *Snapshots of Hemodynamics* 2nd ed. Berlin: Springer (2010).

[14] Convertino, V.A., Doerr, D.F. & Stein, S.L., Changes in size and compliance of the calf after 30 days of simulated microgravity, *J. Appl. Physiol.* **66**, 1509-1512 (1989).

[15] Convertino, V.A., Doerr, D.F., Mathes, K.L., Stein, S.L. & Buchanan, P., Changes in volume, muscle compartment, and compliance of the lower extremities in man following 30 days of exposure to simulated microgravity, *Aviat. Space Environ. Med.* **60**, 653-658 (1989).

[16] Lobachik, V.I., Abrosimov, S.V., Zhidkov, V.V. & Endeka, D.K., Hemodynamic effects of microgravity and their ground-based simulation, *Acta Astronaut.* **23**, 3540 (1991).

[17] Montgomery, L.D., Body volume changes during microgravity II: comparison of horizontal and head-down bed rest, *Aviat. Space Environ. Med.* **64**, 899904 (1993).

[18] Montgomery, L.D., Body volume changes during simulated microgravity: auditory changes, segmental fluid redistribution, and regional hemodynamics, *Ann. Biomed. Eng.* **21**, 417433 (1993).

[19] Noskov, V.B., Redistribution of bodily fluids under conditions of microgravity and in microgravity models, *Hum. Physiol.* **39**, 698706 (2013).

[20] Shoemaker, J.K. et al. Sympathetic discharge and vascular resistence after bed rest, *J. Appl. Physiol.* **84**, 612617 (1998).

[21] van Duijnhoven, N.T.L. et al. The effect of bed rest and an exercise countermeasure on leg venous function, *Eur. J. Appl. Physiol.* **104**, 991998 (2008).

[22] Xiao, X. et al. Bed rest effects on human calf hemodynamics and orthostatic intolerance: a model-based analysis, *Aviat. Space Environ. Med.* **76**, 10371045 (2005).

[23] Buckey, J.C. et al. Orthostatic intolerance after spaceflight, *J. Appl. Physiol.* **81**, 7-18 (1996).

[24] Beckers, F., Verheyden, B., Liu, J. & Aubert, A.E., Cardiovascular autonomic control after short-duration spaceflights, *Acta Astronaut. 65*, 804-812 (2009).

[25] Charles, J.B. et al. Cardiovascular Deconditioning, *NASA Technical Reports Server (NTRS)* 20040201532 (1999).

[26] Di Rienzo, M. et al. Dynamic adaptation of cardiac baroreflex sensitivity to prolonged exposure to microgravity: data from a 16-day spaceflight, *J. Appl. Physiol.* **105**, 15691575 (2008).

[27] Eckberg, D., Halliwill, G.R., Beightol, L.A., Brown, T.E., Taylor, J.A., Human vagal baroreflex mechanisms in space, *J. Physiol.* **588**, 11291138 (2010).

[28] Ertl, A.C. et al. Human muscle sympathetic nerve activity and plasmanoradrenaline kinetics in space, *J. Physiol.* **538**, 321329 (2002).

[29] Migueotte, P.F., Prisk, G.K. & Paiva, M., Microgravity alters respiratory sinus arrhythmia and short-term heart rate variability in humans, *Am. J. Physiol. Heart Circ. Physiol.* 284, H1995H2006 (2003).

[30] Watenpaugh, D.E. et al. Effects of spaceflight on human calf hemodynamics, *J. Appl. Physiol.* **90**, 15521558 (2001).

[31] Henry, W.L., Epstein, S.E., Griffith, J.M., Goldstein, R.E. & Redwood, D.R. Effect of prolonged space flight on cardiac functions and dimensions, In *Biomedical Results from Skylab NASA Technical Reports Server (NTRS)* 19770026836, 366-371 (1977).

[32] Thornton, W.E., Hoffler G.W. & Rummel, J.A., Anthropometric changes and fluid shifts, *NASA Technical Reports Server (NTRS)* 19750006319 (1977).

[33] Thornton, W.E., Moore, T.P. & Pool, S.L., Fluid shifts in weightlessness, *Aviat. Space Environ. Med.* **58**, A8690 (1987).

[34] Arbeille, P. et al. Cardiovascular adaptation to zero-G during a long-term flight (237 days) on board the Salyut-VII soviet space station (1984), *Eur. Space Agency Public* **271**, 134146 (1987).

[35] Moore, T.P. & Thornton, W.E., Space shuttle inflight and postflight fluid shifts measured by leg volume changes, *Aviat. Space Environ. Med.* **58**, A91106 (1987).

[36] Hughson, R.L. et al. Cardiovascular regulation during long-duration spaceflights to the International Space Station, *J. Appl. Physiol.* **112**, 719727 (2012).

[37] Verheyden, B., Liu, J., Beckers, F. & Aubert, A.E., Adaptation of heart rate and blood pressure to short and long duration space missions, *Respir. Physiol. Neurobiol.* **169S**, S13S16 (2009).

[38] Verheyden, B., Liu, J., Beckers, F. & Aubert, A.E., Operational point of neural cardiovascular regulation in humans up to 6 months in space, *J. Appl. Physiol.* **108**, 646654 (2010).

[39] Arbeille, P. et al. Adaptation of the left heart, cerebral and femoral arteries, and jugular and femoral veins during short- and long-term head-down tilt and spaceflight, *Eur. J. Appl. Physiol.* **86**, 157-168 (2001).

[40] Cooke, W.H. et al. Nine months in space: effects on human autonomic cardiovascular regulation, *J. Appl. Physiol.* **89**, 1039-1045 (2000).

[41] Levine, B.D. et al. Human muscle sympathetic neural and haemodynamic responses to tilt following spaceflight, *J. Physiol.* **538**, 331340 (2002).

[42] Zhang, L.F. Region-specific vascular remodeling and its prevention by artificial gravity in weightless environment, *Eur. J. Appl. Physiol.* **113**, 28732895 (2013).

[43] Jarvis, S.S. & Pawelczyk, J.A., The location of the human volume indifferent point predicts orthostatic tolerance, *Eur. J. Appl. Physiol.* **109**, 331341 (2010).

[44] Jarvis, S.S., Pawelczyk, J.A., Identification of the human electrical impedance indifferent point: a surrogate for the volume indifferent point?, *Eur. J. Appl. Physiol.* **107**, 473480 (2009).

[45] Gunga, H.C., Weller von Ahlefeld, V., Appell Coriolano, H.J., Werner, A. & Hoffmann, U., *Cardiovascular System, Red Blood Cells, and Oxygen Transport in Microgravity*, Springer (2016).

[46] Thornton, W.E., Hedge, V., Coleman, E., Uri, J.J. & Moore, T.P., Changes in leg volume during microgravity simulation, *Aviat. Space Environ. Med.* **63**, 789794 (1992).

[47] Bailliart, O. et al. Changes in lower limb volume in humans during parabolic flight, *J. Appl. Physiol.* **85**, 21002105 (1998).

[48] , Petersen, L.G., Damgaard, M., Petersen, J.C. & Norsk, P., Mechanisms of increase in cardiac output during acute weightlessness in humans, *J. Appl. Physiol.* **111**, 407-11 (2011).

[49] Patel, K. et al. Effects of postural changes on cardiovascular parameters across gender, *Medicine* **95**, 28 (2016).

[50] Robertson, D., The pathophysiology and diagnosis of orthostatic hypotension, *Clin. Auton. Res.* **18**, 27 (2008).

[51] Churchill E., Laubach, L.L., Mcconville, J.T. & Tebbetts, I., Anthropometric source book - Volume I: anthropometry for designers, *NASA Technical Reports Server (NTRS)* 19790003563 (1978).

[52] Alfrey, C.P., Udden, M.M., Leach-Huntoon, C., Driscoll, T. & Pickett, M.H., Control of red blood cell mass in spaceflight, *J. Appl. Physiol.* **81**, 98104 (1996).

[53] Norsk, P., Blood pressure regulation IV: adaptive responses to weightlessness, *Eur. J. Appl. Physiol.* **114**, 481497 (2014).

[54] Clément, G., *Fundamentals of Space Medicine*, Springer (2011).

[55] Buckey, J.C. Jr, *Space Physiology*, Oxford University Press (2006).

[56] Martin, D.S., South, D.A., Wood, M.L., Bungo, M. & Fritsch-Yelle, J.M. Comparison of echocardiographic measurements before and after short and long duration spaceflight, *NASA Technical Reports Server (NTRS)* 20100029745 (2000).

[57] Martin, D.S., South, D.A., Wood, M.L., Bungo, M. & Meck, J.V., Comparison of echocardiographic changes after short- and long-duration spaceflight, *Aviat. Space Environ. Med.* **73**, 532-536 (2002).

[58] Aubert, A.E., Beckers, F. & Verheyden, B., Cardiovascular function and basics of physiology in microgravity, *Acta Cardiol.* **60**, 129151 (2005).

[59] Williams, D., Kuipers, A., Mukai, C., Thirsk, R., Acclimation during space flight: effects on human physiology, *CMAJ* **180**, 13171323 (2009).

[60] Perhonen, M.A. et al. Cardiac atrophy after bed rest and spaceflight. *J. Appl. Physiol.* **91**, 64553 (2001).

[61] Abdullah, S.M. et al. Effects of prolonged space flight on cardiac structure and function, *Circulation* **128**, A18672 (2013).

[62] Hughson, R.L., Helm, A. & Durante, M., Heart in space: effects of the extraterrestrial environment on the cardiovascular system, *Nat. Rev. Cardiology* **15**, 167180 (2018).

[63] Herault, S. et al. Cardiac, arterial and venous adaptation to weightlessness during 6-month MIR space flights with and without thigh cuffs (bracelets), *Eur. J. Appl. Physiol.* **81**, 384-390 (2000).

[64] Grigoriev, A.I., Kotovskaya, A.R. & Fomina, G.A., The human cardiovascular system during space flight, *Acta Astronaut.* **68**, 14951500 (2011).

[65] Kotovskaya, A.R. & Fomina G.A., Human venous hemodynamics in microgravity and prediction of orthostatic tolerance in flight, *Hum. Physiol.* **41**, 699-703 (2015).

[66] Fortrat, J.O., de Holanda, A., Zuj, K., Gauquelin-Koch, G. & Gharib, C., Altered venous function during long-duration spaceflights, *Front. Physiol.* **8**, 694 (2017).

[67] Zhu, H., Wang, H. & Liu, Z., Effects of real and simulated weightlessness on the cardiac and peripheral vascular function on humans: a review, *Int. J. Occup. Med. Environ. Health* **28**, 793802 (2015).

[68] Fomina, G.A., Kotovskaya, A.R., Pochuev, V.I. & Zhernavkov, A.F., Mechanisms of changes in human hemodynamics under the conditions of microgravity and prognosis of postflight orthostatic stability, *Hum. Physiol.* **34**, 343-347 (2008).

[69] Fomina, G.A., Kotovskaya, A.R. & Temnova, E.V., Dynamics of human cardiovascular responses in different periods of longterm exposure to weightlessness, *Hum. Physiol.* **38**, 715720 (2012).
